# Disulfide-crosslink analysis of the ubiquitin ligase Hrd1 complex during endoplasmic reticulum-associated protein degradation

**DOI:** 10.1016/j.jbc.2022.102373

**Published:** 2022-08-13

**Authors:** Rudolf Pisa, Tom A. Rapoport

**Affiliations:** Howard Hughes Medical Institute and Department of Cell Biology, Harvard Medical School, Boston, Massachusetts, USA

**Keywords:** ERAD, retrotranslocation, crosslinking, disulfide, Hrd1, Der1, Hrd3, Usa1, Yos9, Bpa, p-benzoyl-L-phenylalanine, ER, endoplasmic reticulum, ERAD, ER-associated protein degradation, DHFR, *E. coli* dihydrofolate reductase, DTT, dithiothreitol, MRH, mannose 6-phosphate receptor homology, sCPY^∗^, a shortened version of CPY^∗^, TM, transmembrane segment

## Abstract

Misfolded proteins in the lumen of the endoplasmic reticulum (ER) are retrotranslocated into the cytosol and degraded by the ubiquitin-proteasome system, a pathway termed luminal ER-associated protein degradation. Retrotranslocation is mediated by a conserved protein complex, consisting of the ubiquitin ligase Hrd1 and four associated proteins (Der1, Usa1, Hrd3, and Yos9). Photocrosslinking experiments provided preliminary evidence for the polypeptide path through the membrane but did not reveal specific interactions between amino acids in the substrate and Hrd1 complex. Here, we have used site-specific disulfide crosslinking to map the interactions of a glycosylated model substrate with the Hrd1 complex in live *S. cerevisiae* cells. Together with available electron cryo-microscopy structures, the results show that the substrate interacts on the luminal side with both a groove in Hrd3 and the lectin domain of Yos9 and inserts a loop into the membrane, with one side of the loop interacting with the lateral gate of Der1 and the other with the lateral gate of Hrd1. Our disulfide crosslinking experiments also show that two Hrd1 molecules can interact through their lateral gates and that Hrd1 autoubiquitination is required for the disassembly of these Hrd1 dimers. Taken together, these data define the path of a polypeptide through the ER membrane and suggest that autoubiquitination of inactive Hrd1 dimers is required to generate active Hrd1 monomers.

Newly synthesized luminal ER proteins undergo quality control to ensure that only properly folded proteins become resident in the ER or are moved on along the secretory pathway (for reviews, see (1, 2, 3, 4, 5, 6)). When a protein cannot reach its native folded state, it is ultimately retrotranslocated into the cytosol, polyubiquitinated, and degraded by the proteasome. This pathway is referred to as luminal ER-associated protein degradation (ERAD-L) and is conserved in all eukaryotes. Work in *S. cerevisiae* showed that ERAD-L requires the Hrd1 complex, consisting of the RING-finger ubiquitin ligase Hrd1 (7, 8, 9) and four additional proteins (Hrd3, Usa1, Der1, and Yos9), each of which is conserved in higher organisms (10, 11).

Recent electron cryo-microscopy (cryo-EM) structures have further clarified the architecture of the Hrd1 complex. The arrangement of Hrd3 and Yos9 suggests that they jointly create a luminal binding site for glycosylated substrates (12). The mannose 6-phosphate receptor homology (MRH) domain of Yos9 binds a terminal α1,6-mannose residue in the glycan, which is generated by glycosidases if a misfolded glycoprotein lingers too long in the ER (13, 14, 15, 16, 17, 18). In addition, an adjacent unstructured substrate segment is thought to bind into a groove of Hrd3 (11, 12, 19). The cryo-EM structures also suggested that the multispanning membrane proteins Hrd1 and Der1 provide the path for the polypeptide chain through the membrane (12). Hrd1 contains eight transmembrane (TM) segments and has a large cytosolic cavity within the membrane. Der1 is an enzymatically inactive member of the rhomboid protease family that possesses six TM segments and a luminal cavity (20, 21). Hrd1 and Der1 do not strongly interact with one another but are linked by Usa1 (10, 22, 23). In addition, Usa1 can oligomerize Hrd1 (10, 22, 23), but Usa1-dependent Hrd1 dimers are not required for ERAD-L (12), and the significance of these oligomers therefore remains unclear. Hrd1 and Der1 both have lateral gates that face one another in a membrane region that is considerably thinner than a normal lipid bilayer (12). Based on the structure, we have proposed that Hrd1 and Der1 form two “half-channels” with cytosolic and luminal cavities, respectively, into which a luminal substrate inserts as a loop (12). Translocation of the tip of the substrate loop would occur through the thinned membrane region between Der1 and Hrd1 (3). This model is supported by site-specific photocrosslinking experiments with probes incorporated into a model substrate (22, 24) or into either Hrd1 (12) or Der1 (25). In these experiments, the position of the photoreactive probe is defined by that of an amber codon, which is suppressed with a modified tRNA charged with p-benzoyl-L-phenylalanine (Bpa) (26, 27). However, the target of the crosslinking reaction is only defined at the protein level; the amino acids of the target protein that are in proximity to the photoreactive probe remain unknown. Identification of the interacting amino acids in both substrate and Hrd1 complex is required to determine the precise path of an ERAD-L substrate.

Studies with overexpressed Hrd1 *in vivo* and with purified protein *in vitro* indicated that Hrd1 polyubiquitinates not only ERAD-L substrates but also itself (28, 29, 30, 31). Autoubiquitination leads to the degradation of Hrd1 *in vivo* (28, 29, 31), but there is also evidence that it activates Hrd1 for ERAD-L *in vitro* and *in vivo* (28, 29, 30). How autoubiquitination would generate active Hrd1 molecules is unknown.

Here, we report on disulfide crosslinking experiments to determine how a substrate moves from the luminal to the cytosolic side of the Hrd1 complex and how autoubiquitination affects Hrd1's activity in ERAD-L.

## Results

### Disulfide crosslinks between a model substrate and Hrd1

**Figure 1:**
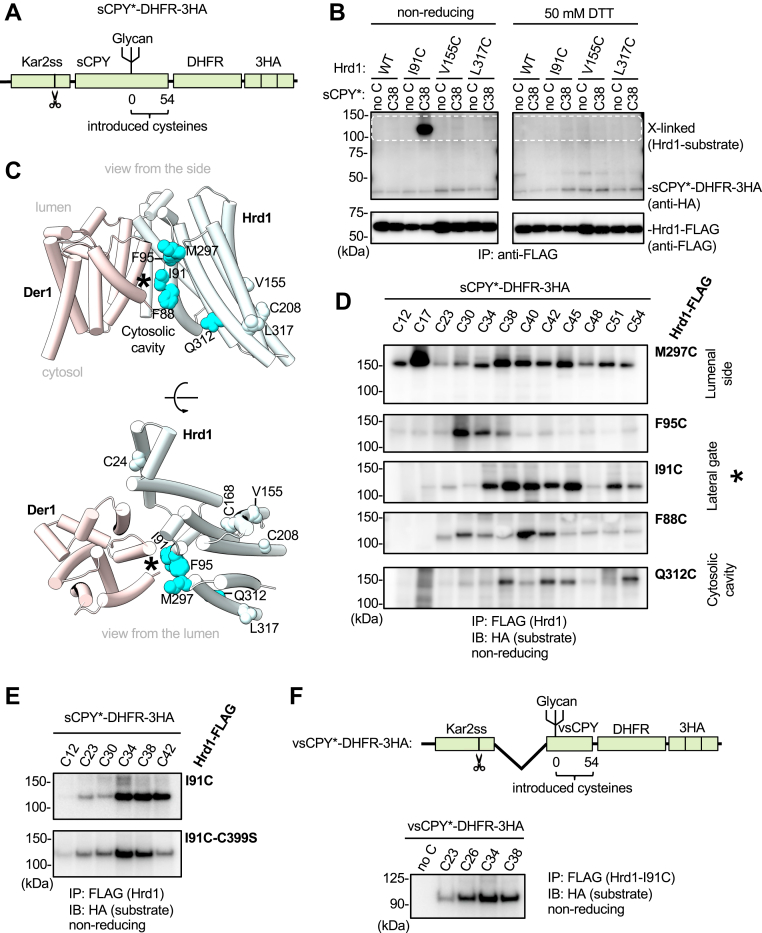
**Probing substrate-Hrd1 interactions by disulfide crosslinking in live cells.***A*, scheme of the model substrate (sCPY^∗^-DHFR-3HA) used for disulfide crosslinking with Hrd1-FLAG. The construct contains the signal sequence of *S. cerevisiae* Kar2 (Kar2ss; the cleavage site is indicated by scissors), a shortened version of CPY^∗^ (sCPY^∗^), *E. coli* dihydrofolate reductase (DHFR), and three hemagglutinin tags (3HA). The different domains are not drawn to scale. Numbers indicate amino acid positions downstream of the glycan at which cysteines were introduced. *B*, a single cysteine was introduced into sCPY^∗^-DHFR-3HA at position +38. The substrate was co-expressed in *hrd1Δ* cells with either wildtype (WT) Hrd1-FLAG or with mutants containing cysteines introduced at the indicated positions. The cells were treated with the oxidant 4,4′-dithiopyridine, and lysates were subjected to immunoprecipitation with anti-FLAG beads, followed by nonreducing (left side of the blots) or reducing (50 mM DTT; right side) SDS-PAGE and immunoblotting with HA and FLAG antibodies. Crosslinked products are visible in the region of the HA blot indicated by white broken lines. The positions of noncrosslinked substrate and Hrd1-FLAG are indicated. *C*, cartoon model of the Hrd1–Der1 complex (PDB code: 6VJZ; Hrd1—*white*, Der1—*pink*) in a side view (upper panel) and a view from the ER lumen (bottom panel). Positions in Hrd1 that gave crosslinks to substrate are shown in ball presentation and colored in *cyan*. Positions that did not crosslink are shown in *white*. The lateral gate of Hrd1 is indicated by a *star*. *D*, As in (*B*), but with sCPY^∗^-DHFR-3HA containing single cysteines at the indicated positions and with Hrd1-FLAG containing cysteines at different positions of the lateral gate. *E*, as in (*D*), but with Hrd1-FLAG-I91C that is either active in polyubiquitination or carries an inactivating mutation in the RING domain (C399S). *F*, As in (*D*), but with a version of the substrate lacking a segment upstream of the glycan attachment site (“very short”—vsCPY^∗^-DHFR-3HA; see scheme). Crosslinking was probed with Hrd1-FLAG-I91C.

We used as a model substrate a shortened version of carboxypeptidase Y^∗^ (sCPY^∗^), a well-characterized misfolded ERAD-L substrate (22). sCPY^∗^ was fused to dihydrofolate reductase (DHFR) and three hemagglutinin (HA) tags (sCPY^∗^-DHFR-3HA). The folded DHFR moiety slows the translocation of the C terminus from the ER lumen into the cytosol, thus increasing the chances of capturing the substrate during its transit through the membrane (13). sCPY^∗^-DHFR-3HA contains only one glycan chain that is trimmed to generate a terminal α1,6-mannose residue for Yos9 recognition (32, 33). Previous photocrosslinking experiments have shown that the strongest interactions of sCPY^∗^-DHFR-3HA with the components of the Hrd1 complex occur with positions downstream of the glycan attachment site (22), which is therefore designated as position "0" (positions downstream are given positive numbers; see scheme in Figure 1*A*). To perform site-specific disulfide crosslinking, we first removed all cysteines from sCPY^∗^-DHFR-3HA and then introduced single cysteines at different positions downstream of the glycan attachment site. In all experiments, the sCPY^∗^-DHFR-3HA mutants were expressed in *S. cerevisiae* cells from a CEN plasmid under the endogenous CPY promoter.

We first tested the interaction of our model substrate with Hrd1. Wildtype Hrd1 or Hrd1 with introduced single cysteines were expressed from a CEN plasmid under the endogenous Hrd1 promoter. Yeast cells expressing both HA-tagged substrate and FLAG-tagged Hrd1 were treated for a short time period with an oxidant (4,4′-dithiopyridine) to induce disulfide bridge formation in live cells. In a pilot experiment, cysteines were introduced at position +38 of sCPY^∗^-DHFR-3HA and position 91 of Hrd1-FLAG (Hrd1-I91C), as Bpa incorporated at these positions showed efficient photocrosslinks (12, 22). After oxidant treatment and quenching, cell lysates were generated, and Hrd1-FLAG was precipitated with beads containing anti-FLAG antibodies. The bound material was analyzed by nonreducing SDS-PAGE, followed by immunoblotting with HA antibodies. A strong band at the expected position for an adduct between sCPY^∗^-DHFR-3HA and Hrd1-FLAG was observed (Fig. 1*B*). The band disappeared after reduction of the disulfide bond with dithiothreitol (DTT) and was not observed with cysteine-less sCPY^∗^-DHFR-3HA or with only one of the two proteins containing the introduced cysteine (Fig. 1*B*). Thus, the two chosen positions of substrate and Hrd1 are in close enough proximity to form a disulfide bridge. Cryo-EM structures indicate that the substrate-interacting Hrd1 residue 91 is located at the lateral gate (Fig. 1*C*). The specificity of the substrate-lateral gate interaction is supported by the fact that no crosslinks were observed with wildtype Hrd1-FLAG, which contains three endogenous cysteines that are outside the lateral gate, but located within the membrane (positions C24, C168, and C208; Fig. S1, *A* and *B*). Furthermore, additional cysteines introduced into the backside of Hrd1 (positions V155, L317) did not give crosslinks (Fig. 1*B*).

To test whether other residues at the lateral gate also crosslink to substrate, we systematically placed cysteines at different positions of Hrd1 (Figs. 1*D* and S1, *A* and *B*). Indeed, efficient disulfide bridge crosslinking was observed with positions on the luminal side of the lateral gate (M297), the membrane-embedded section (F95, I91, and F88), and the cytosolic side (Q312) (Fig. 1*C*). In each case, multiple positions of the substrate formed disulfide bridges with a given cysteine in Hrd1, particularly with the cysteine on the luminal side. None of the substrate cysteines crosslinked to the endogenous cysteines of wildtype Hrd1 (Fig. S1, *A* and *B*). Taken together, these results indicate that the substrate adopts a transmembrane topology along the lateral gate of Hrd1.

The ubiquitination activity of Hrd1 is not required for substrate binding to the lateral gate, as mutation of the essential cysteine in the RING domain (C399S) or the absence of the ubiquitin-conjugating enzyme Ubc7 had little effect on disulfide crosslinking (Figs. 1*E* and S1*C*). Deletion of Der1 also did not abolish substrate crosslinking to Hrd1 (Fig. S1*D*). Although substrate is not degraded in the *der1Δ* strain (Fig. S1*E*), it can still interact with Hrd1, in agreement with experiments in which purified substrate and Hrd1 were studied *in vitro* (30, 34). Importantly, deletion of a CPY^∗^ segment upstream of the glycan attachment site did not affect substrate crosslinking to the lateral gate of Hrd1 (Fig. 1*F*), confirming that only the downstream region of the model substrate is involved in the interaction with the Hrd1 complex.

### Disulfide crosslinks between two Hrd1 molecules

**Figure 2:**
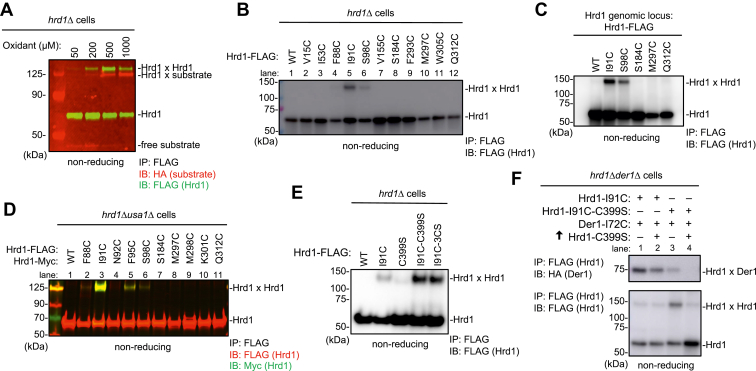
**Lateral gate interaction of Hrd1 molecules.***A*, Hrd1-FLAG-I91C and sCPY^∗^-DHFR-3HA-C38 were co-expressed from CEN plasmids in *hrd1Δ* cells. The cells were treated with increasing concentrations of the oxidant 4,4′-dithiopyridine, and lysates were subjected to immunoprecipitation with anti-FLAG beads, followed by nonreducing SDS-PAGE and immunoblotting with HA and FLAG antibodies. Note the generation of both Hrd1-substrate and Hrd1–Hrd1 disulfide crosslinks. *B*, as in (*A*), but with Hrd1-FLAG–containing cysteines introduced at the indicated positions. The substrate was omitted in these experiments. *C*, as in (*B*), but with Hrd1-FLAG wild-type (WT) or cysteine mutants expressed from the genomic locus of Hrd1. *D*, as in (*B*), but with co-expression of differently tagged Hrd1 versions (Hrd1-FLAG and Hrd1-Myc), each with cysteines introduced at the indicated positions. Both Hrd1 versions were expressed from CEN plasmids in *hrd1Δusa1Δ* cells. *E*, as in (*B*), but with Hrd1-FLAG-I91C that is either active in polyubiquitination or carries inactivating mutations in the RING domain (C399S or C377S, C396S, C399S - denoted as 3CS). *F*, as in (*B*), but co-expressing Der1-HA-I72C and either Hrd1-FLAG-I91C or Hrd1-FLAG-I91C-C399S in *hrd1Δder1Δ* cells from CEN plasmids under the endogenous promoters. Where indicated, Hrd1-C399S was overexpressed from a 2μ plasmid and the TDH3 promoter. sCPY^∗^, a shortened version of CPY^∗^; DHFR, *E. coli* dihydrofolate reductase; 3HA, three hemagglutinin tags.

In addition to the substrate–Hrd1 crosslinks, we observed Hrd1–Hrd1 disulfide bridge formation with cysteines introduced at the lateral gate (I91) (Figs. 2*A* and S2*A*). These crosslinks were observed in the presence (Fig. 2*A*) or absence (Figs. 2*B* and S2*B*) of the model substrate and occurred with another residue at the lateral gate (S98) (Fig. 2*B*), even when Hrd1 was expressed at endogenous levels (Figs. 2*C* and S2*C*). The identity of the crosslinks was confirmed by using Hrd1 tagged with different epitopes (Figs. 2*D* and S2*D*). Cysteines introduced at positions outside the lateral gate did not give these crosslinks (Fig. 2, *B* and *D*). Because previous experiments indicated that Usa1 can mediate Hrd1 oligomerization (10, 23), we tested disulfide crosslinking in a *usa1Δ* strain (Figs. 2*D* and S2*E*). No differences to wildtype cells were observed (Fig. 2*D*), indicating that the Hrd1–Hrd1 interaction across the lateral gates is independent of Usa1. Hrd1–Hrd1 crosslinks were also unaffected by the deletion of *DER1* (Fig. S2*E*), whereas they increased in the absence of *UBC7* (Fig. S2*E*), suggesting that polyubiquitination is required to dissolve the observed Hrd1 dimers. This interpretation is supported by the observation that the intensity of the Hrd1–Hrd1 crosslinks was considerably increased when Hrd1 carried mutations in the RING domain, which abolish its ubiquitination activity (Figs. 2*E* and S2*F*). The high crosslinking efficiency suggested that Der1 is replaced by Hrd1 at its lateral gate position. Indeed, the overexpression of the ubiquitination-inactive Hrd1 mutant in a strain containing active Hrd1 led to the reduction of Hrd1–Der1 crosslinking (Fig. 2*F*, lane 2 *versus* 1). Furthermore, cells expressing exclusively the inactive Hrd1 mutant gave only weak Hrd1–Der1 crosslinks (lane 3), and these crosslinks were completely abolished when the inactive mutant was overexpressed (lane 4). These data suggest that autoubiquitination of Hrd1 disassembles Hrd1 dimers and instead allows the formation of the Hrd1–Der1 complex. Consistent with this assumption, Hrd1–Der1 crosslinks were reduced in favor of Hrd1–Hrd1 crosslinks when autoubiquitination was prevented by mutating all Lys residues to Arg in the RING finger of Hrd1 (KRK mutant (28); Fig. S2*G*).

### Disulfide crosslinks between substrate and Der1

**Figure 3:**
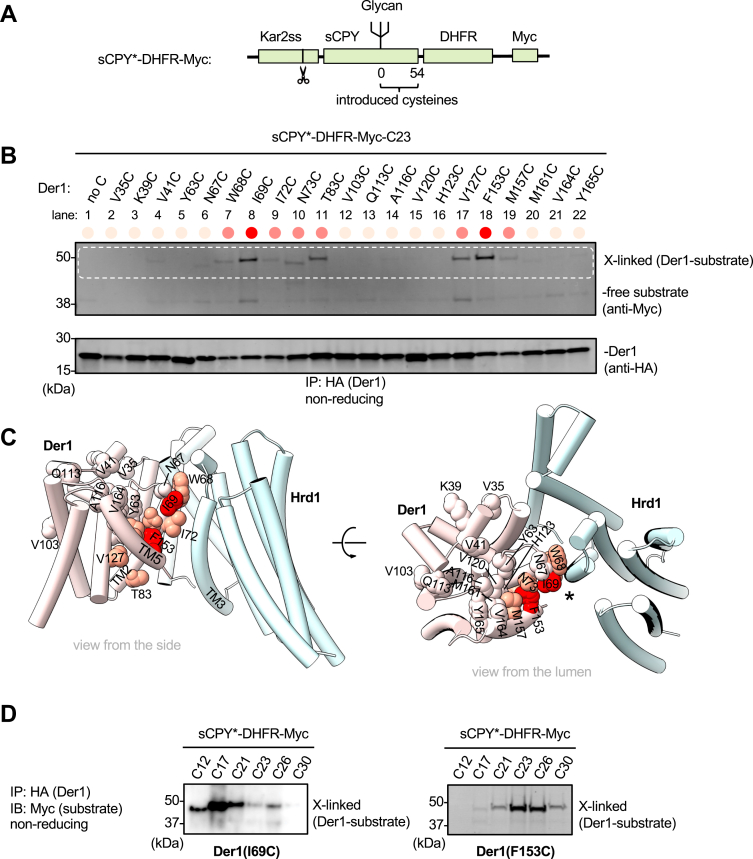
**Probing substrate-Der1 interactions by disulfide crosslinking.***A*, scheme of the model substrate containing a Myc epitope at the C terminus (sCPY^∗^-DHFR-Myc). *B*, sCPY^∗^-DHFR-Myc with a cysteine at position +23 was co-expressed with Der1-HA constructs containing single cysteines introduced at the indicated positions. The substrate and Der1-HA were expressed from CEN plasmids in *der1Δ* cells. The cells were treated with the oxidant 4,4′-dithiopyridine, and lysates were subjected to immunoprecipitation with anti-HA beads, followed by nonreducing SDS-PAGE and immunoblotting with HA and Myc antibodies. Crosslinked products appear in the indicated region of the blot (*white broken line*). The positions of noncrosslinked substrate and Der1-HA are indicated. *C*, cartoon model of the Hrd1–Der1 complex (PDB code: 6VJZ; Hrd1—*light cyan*, Der1—*pink*) in views from the side (left panel) and from the ER lumen (right panel). Der1 positions that gave crosslinks to substrate are indicated in *orange* and *red*, with red indicating high efficiency of crosslinking. Positions that did not crosslink are shown in *white*. The lateral gate of Der1 is indicated by a star in the right panel. *D*, as in (*B*), but with Der1 carrying cysteines at positions I69 or F153 and sCPY^∗^-DHFR-Myc with cysteines at the indicated positions. sCPY^∗^, a shortened version of CPY^∗^; DHFR, *E. coli* dihydrofolate reductase; TM, transmembrane.

Next, we tested the interaction of our model substrate with Der1. In this case, we expressed Der1 with a single HA tag at the C terminus and used sCPY^∗^-DHFR tagged with a Myc epitope (sCPY^∗^-DHFR-Myc; Figure 3*A*). We initially introduced a cysteine at position +23 of this substrate, as a Bpa probe at this position gave strong photocrosslinks to Der1 (22). The tested cysteines in Der1 were placed primarily at the lateral gate, *i.e.*, at TMs 2 and 5, but also at various control positions. The cells were treated with the oxidant 4,4′-dithiopyridine, quenched, and cell lysates were subjected to immunoprecipitation with beads containing HA antibodies. Bound material was analyzed by SDS-PAGE and immunoblotting with Myc antibodies (Fig. 3*B*). Strong disulfide crosslinks were only observed with positions at the lateral gate of Der1 (Fig. 3, *B* and *C*; approximate crosslinking yields are shown qualitatively in shades of red). The most prominent interaction with substrate was seen with residues inside the membrane (Fig. 3*B*, and *C*, dark red). As expected, the crosslinks disappeared after DTT treatment (Fig. S3*A*) and were dependent on the presence of Hrd1 (Fig. S3*B*). All positions outside the lateral gate of Der1 did not crosslink (Fig. 3*C*; residues shown in *white*). Next, we chose several cysteine Der1 mutants and varied the position of the cysteine in sCPY^∗^-DHFR-Myc. Substrate cysteines located between +12 and +26 crosslinked efficiently to the lateral gate of Der1 (Figs. 3*D* and S3*C*). Position +12 and +17 of the substrate crosslinked most strongly to cysteines at the luminal side of Der1 (positions A116, Y165, and I69), whereas position +26 crosslinked to the cytosolic side (T83) (Fig. S3*C*). Position +30 crosslinked only weakly or not at all to the chosen Der1 positions (Fig. S3*C*). Taken together, these data indicate that the substrate specifically contacts the lateral gate of Der1 and that the Der1-interacting region of the substrate is closer to the glycan-attachment site than the one contacting Hrd1.

### Disulfide crosslinks between Hrd1 and Der1

**Figure 4:**
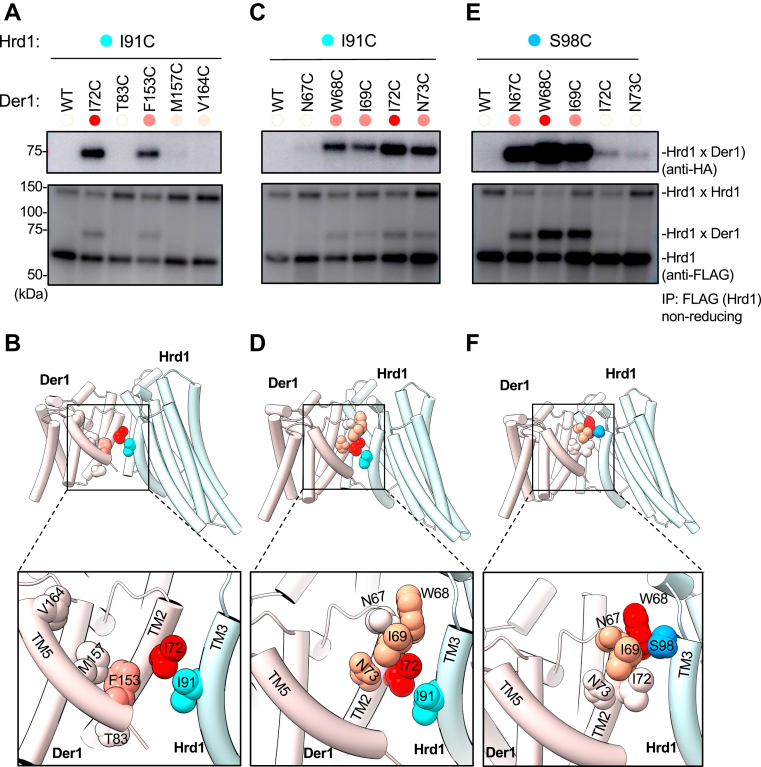
**Probing Hrd1-Der1 proximity by disulfide crosslinking.***A*, Hrd1-FLAG-I91C was co-expressed with Der1-HA containing single cysteines at the indicated positions in TM2 and TM5. The cells were treated the oxidant 4,4′-dithiopyridine, and lysates were subjected to immunoprecipitation with anti-FLAG beads, followed by nonreducing SDS-PAGE and immunoblotting with HA and FLAG antibodies. The efficiency of crosslinking is qualitatively indicated by the shades of red of the dots above the lanes. *B*, cartoon model of the Hrd1-Der1 complex viewed from the side (PDB code: 6VJZ; Hrd1—*light cyan*, Der1—*pink*). The lower panel shows a zoomed-in view. Der1 positions that crosslink to substrate are indicated with the efficiency of crosslinking indicated by shades of *red*. Positions that did not crosslink are shown in *white*. Hrd1 position I91 is highlighted in *cyan*. *C*, as in (*A*), but with additional cysteine positions in Der1-HA. *D*, as in (*B*), but with the positions chosen in (*C*). *E*, as in (*C*), but with the cysteine in Hrd1 at position S98 (Hrd1-FLAG-S98C). *F*, as in (*D*), but with Hrd1-FLAG-S98C. S98 is highlighted in *blue*. TM, transmembrane.

The substrate-interacting lateral gate residues of Hrd1 and Der1 also formed disulfide crosslinks with each other. When a cysteine was introduced at position I91 of Hrd1, it efficiently formed a disulfide bridge with residues in the lateral gate helices TM2 and TM5 of Der1 (Fig. 4, *A* and *B*). A more detailed analysis showed that I91 located in TM3 of Hrd1 crosslinked best with residue I72 of Der1's TM2 (Fig. 4, *C* and *D*). This interaction was highly specific, as moving the cysteine to residue S98 in TM3 of Hrd1, *i.e*., by two helical turns toward the luminal side, abolished crosslinking to Der1's I72 position (Fig. 4, *E* and *F*); instead, S98C now crosslinked most efficiently with the W68C of Der1, which is also closer to the ER lumenal side. These results are consistent with the structural model of the Hrd1/Der1 complex derived from cryo-EM data (12) (Fig. 4, *B* and *D* and *F*), as well as with photocrosslinking data (12) and indicate that the lateral gates of the two proteins face each other and the substrate.

### Disulfide crosslinks between substrate and Hrd3

**Figure 5:**
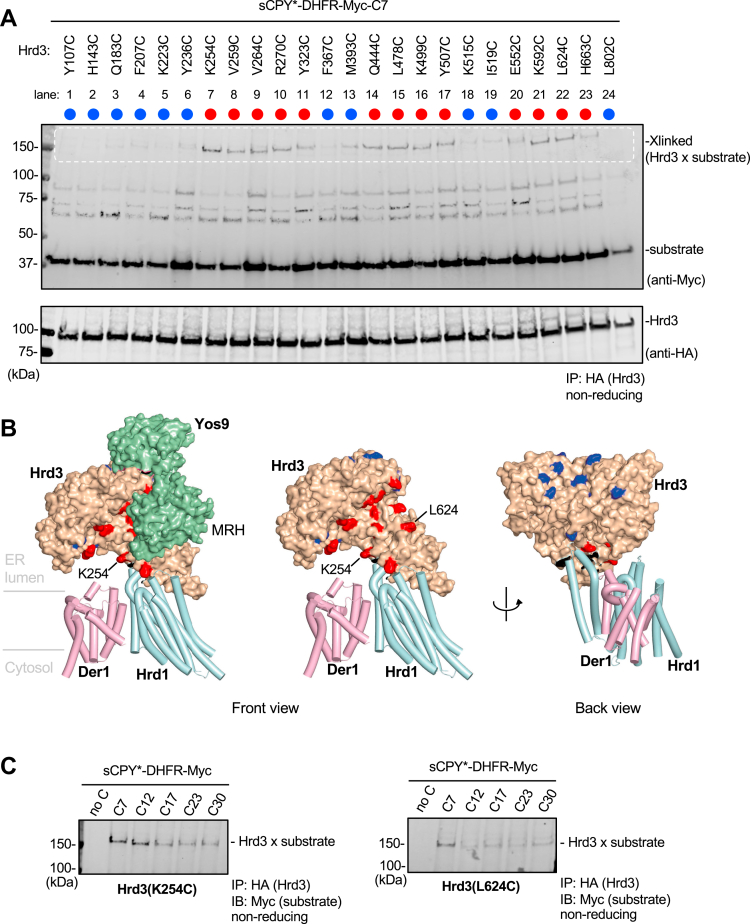
**Probing Hrd3-substrate interactions by disulfide crosslinking.***A*, sCPY^∗^-DHFR-Myc with a cysteine at position +7 was co-expressed with 3HA-Hrd3 containing single cysteines introduced at the indicated positions. The substrate and 3HA-Hrd3 were expressed from CEN plasmids in *hrd3Δ* cells. The cells were treated with the oxidant 4,4′-dithiopyridine, and lysates were subjected to immunoprecipitation with anti-HA antibody beads, followed by nonreducing SDS-PAGE and immunoblotting with HA and Myc antibodies. Crosslinked products appear in the indicated region of the blot (*white broken line*). The positions of noncrosslinked substrate and 3HA-Hrd3 are indicated. *B*, model of the Hrd1-Der1-Hrd3-Yos9 complex in three different side views. Der1 and Hrd1 are shown as cartoons, Hrd3 and Yos9 as space-filling models. The overall model is based on two structures, one containing a fusion of Hrd1 and Usa1, Der1, and Hrd3 (PDB code: 6VJZ) and the other containing Hrd3 and Yos9 (PDB code: 6VK3); Hrd3 was used as a template to align the two structures. Hrd1, Der1, Hrd3, and Yos9 are shown in *light cyan*, *pink*, *light brown*, and *green*, respectively. Hrd3 positions that gave crosslinks to substrate are indicated in *red*. Positions that did not crosslink are shown in *blue*. MRH—mannose 6-phosphate receptor homology domain of Yos9. *C*, as in (*A*), but with Hrd3 carrying cysteines at positions K254 or L624 and sCPY^∗^-DHFR-Myc with cysteines at the indicated positions. sCPY^∗^, a shortened version of CPY^∗^; DHFR, *E. coli* dihydrofolate reductase.

To analyze the interaction of substrate with Hrd3, we attached three HA tags to the N terminus of Hrd3 and again used sCPY^∗^-DHFR-Myc as substrate. Based on previous photocrosslinking experiments (22, 24), we initially placed a cysteine at position +7 of the substrate. The cysteine in Hrd3 was placed at positions facing Hrd1, Der1, or Yos9, as well as at several positions pointing away from them. The results of the crosslinking experiments, performed as described for Der1, show that the strongest substrate interaction occurred with Hrd3 positions close to a groove observed in cryo-EM structures (12) (Fig. 5, *A* and *B*; positions in red; DTT control shown in Fig. S4*A*). This groove is adjacent to the glycan-binding MRH domain of Yos9 and faces Hrd1 and Der1. Some of the crosslinking positions, including L624, are covered by the MRH domain in the cryo-EM structure, but the MRH domain seems to be flexible and may transiently dissociate (12). No crosslinks were seen with several positions distant from the groove (Fig. 5, *A* and *B*; positions in blue).

Next, we chose two Hrd3 mutants with cysteines in the groove (K254C and L624C) and varied the position of the cysteine in sCPY^∗^-DHFR-Myc. As expected, no crosslinks were observed with cysteine-less sCPY^∗^-DHFR-Myc (Figs. 5*C* and S4*B*). Substrate with cysteine at position +7 crosslinked most efficiently to both Hrd3 mutants. Position +12 of the substrate also crosslinked well to K254C (Fig. 5, *B* and *C*), a position further away from the glycan-interacting MRH domain than L624C. Positions +17 to +30 crosslinked only weakly or not at all to the chosen Hrd3 positions (Fig. 5*C*). Thus, the groove seems to accommodate a substrate segment that is close to the glycan attachment site.

## Discussion

**Figure 6:**
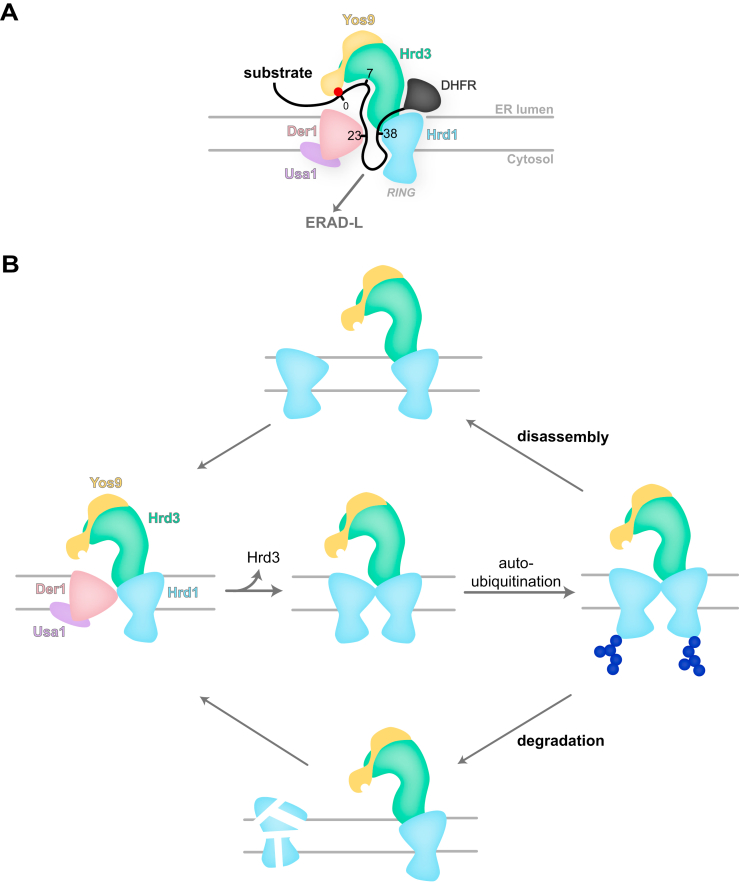
**Models for substrate insertion into the Hrd1 complex and for activation of Hrd1 for ERAD-L.***A*, scheme of the Hrd1 complex with inserted ERAD-L model substrate. The glycan-attachment site is indicated by a *red dot*. Positions downstream of the glycan that crosslink to the different components of the Hrd1 complex are indicated. The RING domain of Hrd1 is indicated. *B*, Hrd1–Hrd1 dimers, in which the monomers interact across their lateral gates, can form following the dissociation of Hrd3 from the Hrd1 complex. These dimers cannot mediate ERAD-L or -M. Hrd1 autoubiquitination can either cause the disassembly of the Hrd1 dimers into monomers or lead to the degradation of one of the Hrd1 molecules. Following deubiquitination, the surviving Hrd1 molecules can become active participants in ERAD. ERAD-L, luminal ER-associated protein degradation; ERAD-M, ER-associated protein degradation of membrane proteins; DHFR, dihydrofolate reductase.

Our disulfide crosslinking data result in a residue-resolution model for how a glycosylated ERAD-L substrate interacts with the Hrd1 complex (Figs. 1*C* and 3*C* and 5*B* and 6*A*). The terminal α1,6-mannose residue in the substrate-attached glycan chain interacts with the MRH domain of Yos9 and serves as the anchor point (Fig. 6*A*). All interactions with the Hrd1 complex components occur with regions downstream of the glycan attachment site (22, 24), as confirmed here with a deletion mutant of the substrate. We now show that, as proposed (12), the region immediately adjacent to the glycan interacts with a groove in Hrd3. This groove can only accommodate an extended polypeptide segment but does not contain an unusual number of hydrophobic amino acids. Thus, Hrd3 does not recognize the unfolded substrate like a chaperone that binds to hydrophobic amino acids normally buried in a folded protein. Instead, the Hrd3 groove might simply accommodate a flexible polypeptide segment. The dual recognition of the α1,6-mannose residue by the MRH domain and of the adjacent flexible peptide segment by Hrd3 would ensure that only terminally unfolded glycoproteins bind to the Hrd1 complex, while folding intermediates in the ER lumen are ignored.

The substrate segment following the Hrd3/Yos9 interacting region seems to insert into the Der1/Hrd1 complex as a loop (Fig. 6*A*). The disulfide crosslinking data show that the glycan-proximal side of the hairpin specifically interacts with the lateral gate of Der1 (TMs 2 and 5). Our results are more convincing than those of previous photocrosslinking experiments with Bpa probes in Der1 and full-length CPY^∗^, in which most residues gave some crosslinks (25). Structural data for Der1 and for the related rhomboid protein GlpG show that the lateral gate can open by movement of TM5 (12), which in the case of GlpG allows the TM segment of a substrate to enter sideways and be cleaved by active site residues in a luminal cavity (35, 36). The surface of the laterally open Der1 molecule displays only few hydrophobic amino acids, in large part because TM2 contains hydrophilic residues on its cytosolic end, which are important for its function in ERAD (12). Thus, the lipid bilayer in this region is likely thinned and disturbed, which may allow the glycan-proximal segment of the substrate hairpin to adopt a transmembrane orientation, despite the fact that it contains only few hydrophobic amino acids.

The glycan-distal side of the substrate hairpin interacts with the lateral gate of Hrd1 (Fig. 6*A*). This segment contacts the lateral gate all the way from the luminal to the cytosolic side. Again, only a small part of the interacting Hrd1 surface is hydrophobic, so most of the membrane-spanning substrate segment is located in an aqueous milieu. Thus, both Der1 and Hrd1 ensure that only a small number of substrate residues are located in a hydrophobic environment inside the membrane, thus minimizing the energetic costs to establish the transmembrane orientation of the substrate hairpin. In addition, moving the tip of the hairpin across the membrane would be facilitated by the fact that the membrane region between the lateral gates of Der1 and Hrd1 is much thinner than that of a normal lipid bilayer (3, 12). Interestingly, the same residues of Hrd1 and Der1 that interact with substrate are also close to each other. Thus, it is possible that the Hrd1–Der1 interface is flexible or that the two proteins transiently dissociate to accommodate the substrate. Transient dissociation is conceivable given the small interface surface seen in cryo-EM structures (12).

Our disulfide crosslinking experiments show that two Hrd1 molecules can associate across their lateral gates. As expected, such an arrangement sterically blocks the interaction of Hrd1 with Der1. Since Der1 is required for the recruitment of luminal substrates, these Hrd1–Hrd1 dimers must be inactive in ERAD-L. They are probably also unable to handle misfolded membrane proteins (ERAD-M substrates) because the lateral gates of both Hrd1 molecules are mutually blocked. Furthermore, our cryo-EM structures indicate that the two Hrd1 molecules can only interact through their lateral gates when at least one of them is not associated with Hrd3 (12); otherwise, there would be steric clashes. Given that Hrd3 is required to recruit luminal substrates, this again suggests that one or both molecules in the Hrd1 dimer must be inactive. Our results show that ubiquitination activity of Hrd1, specifically its ability to autoubiquitinate, is required to dissolve the Hrd1 dimers. In one possible scenario (Fig. 6*B*), one of the Hrd1 molecules in the dimer would serve as an ERAD substrate for the other. A segment of the "substrate Hrd1" would enter the lateral gate of the neighboring Hrd1 molecule and undergo autoubiquitination and subsequent degradation. Ultimately, the remaining Hrd1 molecule could re-associate with Hrd3 and Der1 and reparticipate in ERAD. In an alternative scenario (Fig. 6*B*), autoubiquitination of the Hrd1 dimer would simply separate the two Hrd1 molecules. Following deubiquitination, both Hrd1 molecules could become active participants in ERAD. This model may explain why autoubiquitination activates Hrd1 *in vitro*, in the absence of protein degradation (28). However, *in vivo*, it seems likely that at least some Hrd1 dimers are intermediates of Hrd1 degradation. Such Hrd1 dimers could be generated by the transient dissociation of Hrd3 from Hrd1 and would explain the relatively short half-live of endogenous Hrd1 (29). In cells lacking Hrd3, there would be no impediment to Hrd1 dimer formation, explaining why Hrd1 is rapidly polyubiquitinated and degraded (31, 37). The degradation of Hrd1 is counteracted by the deubiquitinating enzyme Ubp1, which in turn is inhibited by the UBL domain of Usa1 (29), explaining why Usa1 stimulates Hrd1 degradation (38). In this model, Hrd3 would not only help to recruit luminal substrates but also prevent Hrd1 dimerization, which otherwise would lead to its autoubiquitination and degradation.

## Experimental procedures

### Plasmids and strains

The plasmids used for the expression of Hrd1, Hrd3, Der1, and of CPY-derived substrates were all versions of the low-copy pRS31x or 41x vectors harboring CEN/ARS sequences. All constructs in low-copy vectors were expressed under their respective endogenous promoter and terminator sequences. Full length Hrd1 was tagged with a GSGGASGGSG linker followed by a single FLAG epitope (DYKDDDDK) at its C terminus (12). Der1 was tagged with a single HA epitope (YPYDVPDYA) at the C terminus. Hrd3 was tagged at its N terminus with 3xHA tag as described previously (31). For overexpression of the Hrd1(C399S) from the high-copy (2μ) pRS423 plasmid, the coding sequence was placed under the TDH3 promoter and a CYC1 terminator. Cysteines in sCPY^∗^-DHFR-3HA were removed using PCR-based site-directed mutagenesis (C85 and C152 in *Ec*-DHFR were replaced with alanine and serine (39), respectively and a cysteine present downstream of the 3HA tag was replaced with serine). To generate sCPY^∗^-DHFR-Myc, cysteine-less sCPY^∗^-DHFR-3HA was digested by NheI and BlpI and ligated with a double-stranded DNA fragment containing a GSGGASGGSG linker followed by a single Myc epitope (EQKLISEEDL).

BY4741 wildtype strain and *hrd3Δ*, *der1Δ*, and *ubc7Δ* knockout strains (all MATa) were obtained from Horizon Discovery. BY4741 *hrd1Δubc7Δ* double knockout strain was generated from the BY4741 *ubc7Δ* strain by replacing endogenous *HRD1* coding sequence with a nourseothricin resistance–conferring cassette (NatR). Briefly, cells were transformed using standard Li acetate technique (40) by a PCR product encoding the NatR cassette with 5′ and 3′ flanking regions (∼200 bp) homologous to the *HRD1* promoter and terminator sequences. After heat shock (42 °C, 45 min), cells were recovered in fresh YPD media for ∼3 h at 30 °C and transferred onto YPD plates supplemented with 200 μg/ml nourseothricin sulfate (Cat.No. N-500, GoldBio) and incubated at 30 °C until single colonies emerged. Correct incorporation of the NatR cassette at the Hrd1 locus was confirmed by PCR and Sanger sequencing.

FY251 *hrd1Δ*, FY251 *hrd1Δder1Δ*, and BY4741 *hrd1Δusa1Δ* knockout strains were described previously (10, 12, 22). All strains transformed with plasmids were selected on amino acid drop-out plates in synthetic medium (Teknova). Multiple colonies were picked to inoculate a starter culture (∼4 ml), which was incubated overnight at 30 °C. Larger cultures (50–100 ml) were inoculated by diluting the starter cultures to *A*_600_ ∼0.1 and grown at 30 °C until the *A*_600_ reached 0.5 to 0.8. The cells were then spun at 3000 rpm for 3 min at room temperature, and the cell pellet resuspended in ∼5 ml PBS or medium supplemented with 500 μM 4,4′-dithiopyridine (Aldrithiol-4, Cat.No. 143057, Millipore-Sigma). After 15 min incubation at 30 °C in a shaker (230 rpm), the reactions were quenched with N-ethylmaleimide (final concentration: 20 mM) for 15 min on ice. Cells were then pelleted, flash frozen in liquid nitrogen, and stored at −80 °C for later use.

### Immunoprecipitation and Western blotting

Cell pellets in 2 ml screw-top tubes were thawed on ice and resuspended in ∼1 ml of ice-cold immunoprecipitation buffer (IPB: 25 mM Hepes/NaOH pH 7.4, 200 mM NaCl, 5% glycerol) supplemented with a protease inhibitor cocktail and 1 mM PMSF. Cells were lysed at 4 °C with glass beads (0.5 mm diameter, Cat.No. 11079105, Biospec) in a beadbeater (Mini-Beadbeater-16, Biospec), and cell debris were removed by centrifugation (3000 rpm, 5 min). Membrane fractions were then collected by ultracentrifugation at 50,000 rpm for 30 min at 4 °C (TLA-55 rotor; Optima TLX ultracentrifuge, Beckman Coulter) and flash frozen or used immediately. Membranes were homogenized by pipetting in IPB (∼50 μl) and solubilized with IPB (∼500 μl) containing 1.1% Igepal CA-630 (Cat.No. I3021, Millipore-Sigma) for 1 h on a rotator in the cold room. The extract was then spun at 50,000 rpm for 15 min at 4 °C in a TLA-55 rotor (Beckman Coulter), and the supernatant (∼0.5 ml) was incubated with 7 to 10 μl of appropriate agarose resin or magnetic bead suspension for 2 h at 4 °C. Beads were washed with IPB containing 0.1% Igepal CA-630, and bound protein was eluted and separated by nonreducing SDS-PAGE or treated with 50 mM DTT prior the electrophoresis. Hrd1-FLAG constructs were immunoprecipitated with anti-FLAG M2 resin (EZview, Cat.No. F2426, Millipore Sigma) or Pierce anti-DYKDDDDK magnetic agarose (Cat.No. A36797, ThermoFisher). The bound protein was eluted with IPB containing 0.1% Igepal CA-630 and 0.2 mg/ml 3xFLAG peptide (Cat.No. B23111, Bimake) or eluted by 1× SDS loading buffer supplemented with 8 M urea (1× SDS-U). Der1-HA and 3xHA-Hrd3 constructs were immunoprecipitated with Pierce anti-HA magnetic beads (Cat.No. 88836, ThermoFisher) and eluted with 1× SDS-U. Eluted samples were heated for 10 min at 60 °C (90°C for samples supplemented with 50 mM DTT) prior to SDS-PAGE and immunoblotting. FLAG-tagged proteins were detected using anti-FLAG polyclonal antibody produced in rabbit (Cat.No. F7425, Millipore-Sigma; diluted 1:2000). HA-tagged proteins were detected using anti-HA monoclonal antibody from rat (clone 3F10, Roche, Cat.No. 11867423001, 1:3000). Myc-tagged proteins were detected using anti-Myc tag polyclonal antibody from rabbit (Cat.No. Ab9106, Abcam, 1:3000). Secondary antibodies used for visualization were conjugated to horseradish peroxidase (HRP) or a fluorescent dye (mouse anti-rabbit HRP, Cat.No. SC2357, Santa Cruz Bio; goat anti-rat HRP, Cat.No. 31470, ThermoFisher; IRDye goat anti-rat-800CW Cat.No. 926–32219, Licor; IRDye goat anti-rat-680RD Cat.No. 926–68076, Licor; IRDye donkey anti-rabbit-800CW Cat.No. 926–32213; IRDye donkey anti-rabbit-680RD Cat.No. 926–68073, Licor). All secondary antibodies were diluted 1:10,000.

### Cycloheximide-chase experiments

Cycloheximide-chase degradation assays were performed as described (12). Briefly, yeast cells grown on SC dropout plates were used to inoculate a preculture (∼4 ml) and grown overnight at 30 °C. The cells were diluted next day in 50 ml synthetic dropout media to ∼0.1 *A*_600_/ml and grown in a shaker at 30 °C. When the cultures reached 0.4 to 0.6 *A*_600_/ml, the cells were spun, and the pellet resuspended in ∼10 ml of prewarmed fresh media supplemented with 100 μg/ml cycloheximide (Cat.No. C7698, Millipore-Sigma). A 2 ml aliquot was taken as the “0 min” timepoint, mixed with sodium azide (0.05–0.1%) on ice, spun (10,000 rpm, 1 min), and the pellet was flash frozen in liquid nitrogen. The remaining culture was incubated in a shaker at 30 °C, and samples were taken at indicated timepoints and processed as the “0 min” timepoint. Cell pellets were mixed with ∼250 μl of glass beads (0.5 mm diameter, Cat.No. 11079105, Biospec) and ∼200 μl of lysis buffer (10 mM MOPS, pH 6.8, 1% SDS, 8 M urea, 10 mM EDTA, yeast/fungi protease inhibitor cocktail (Cat.No. GB-333–1, GoldBio)) and lysed by vortexing (2 × 1 min). The lysates were supplemented with 4× SDS loading buffer, incubated at 65 °C for 10 min, spun in a table-top centrifuge (15,000 rpm), and subjected to SDS-PAGE and immunoblotting with anti-HA antibody and anti-Pgk1 antibody (mouse monoclonal [22C5D8], Cat.No. ab113687, AbCam, diluted 1:5000). Goat anti-rat HRP and IRDye goat anti-mouse-680RD (Cat.No. 926–68070, Licor) were used for visualization. Signal intensities were determined in ImageStudio software (Licor) and normalized to the “0 min” timepoint. Means and standard deviations from three independent experiments were calculated in Prism (v9.3.1).

## Data availability

All data are available in the article or its supporting information.

## Supporting information

This article contains supporting information (Figs. S1–S4).

## Conflict of interest

The authors declare that they have no conflicts of interest with the contents of this article.
