# Supplementary material for: Disulfide-crosslink analysis of the ubiquitin ligase Hrd1 complex during endoplasmic reticulum-associated protein degradation
Source: J Biol Chem. 2022 Aug 13;298(9):102373. doi: 10.1016/j.jbc.2022.102373 (PMC9478403; doi:10.1016/j.jbc.2022.102373)
Supplement: Supplemental Figure Legends [file mmc1.docx]

**Supporting Information**

**Figure S1. Systematic disulfide crosslinking of sCPY*-DHFR-3HA and Hrd1-FLAG.**

(**A**) Single cysteines were introduced into sCPY*-DHFR-3HA and Hrd1-FLAG at the indicated positions and co-expressed from CEN plasmids in *hrd1∆* cells (see also **Fig. 1D**). The cells were treated with the oxidant 4,4’‑dithiopyridine and lysates were subjected to immunoprecipitation with anti-FLAG beads, followed by non-reducing (left side of the blots) or reducing (50 mM DTT; right side) SDS-PAGE and immunoblotting with HA antibodies.

(**B**) As in (A), but the blot was analyzed with FLAG antibodies.

(**C**) As in (A), but with *hrd1∆* or *hrd1∆ubc7∆* cells expressing Hrd1-FLAG-I91C from a CEN plasmid and substrate containing a cysteine at position +38. The samples were treated with the oxidant 4,4’‑dithiopyridine, followed by non-reducing SDS-PAGE and immunoblotting with Myc and FLAG antibodies. The asterisks indicate non-specific bands.

(**D**) As in (C), but with *hrd1∆* or *hrd1∆der1∆* cells expressing sCPY*-DHFR-3HA-C38 substrate. The samples were analyzed by non-reducing SDS-PAGE and immunoblotting with HA and FLAG antibodies.

(**E**) The degradation of sCPY*-DHFR-3HA-C38 was tested in cycloheximide-chase experiments using cells lacking the indicated genes and expressing Hrd1-FLAG-I91C from a CEN plasmid. The left panel shows a representative HA blot. Blotting for phosphoglycerate kinase 1 (Pgk1) was used as a loading control. The right panel shows the quantification of three independent experiments (signs and error bars represent means and standard deviations, respectively).

**Figure S2.** **Probing the lateral gate interactions of Hrd1-molecules.**

(**A**) Control for the experiment in **Fig. 2A**. Hrd1-FLAG-I91C and sCPY*-DHFR-3HA-C38 were co-expressed from CEN plasmids in *hrd1∆* cells. The cells were treated with increasing concentrations of the oxidant 4,4’‑dithiopyridine and lysates were subjected to immunoprecipitation with anti-FLAG beads. The samples were treated with 50 mM DTT prior SDS-PAGE and immunoblotting with HA and FLAG antibodies.

(**B**) Control for the experiment in **Fig. 2B**. The samples were treated with 50 mM DTT prior SDS-PAGE and immunoblotting.

(**C**) Control for the experiment in **Fig. 2C**. The samples were treated with 50 mM DTT prior SDS-PAGE and immunoblotting.

(**D**) Control for the experiment in **Fig. 2D**. The samples were treated with 50 mM DTT prior SDS-PAGE and immunoblotting.

(**E**) Hrd1-FLAG-I91C was expressed in *hrd1∆der1∆, hrd1∆usa1∆ or hrd1∆ubc7∆* cells from a CEN plasmid together with an empty vector (e.v.) or a CEN plasmid coding for Der1, Usa1, or Ubc7, each under its endogenous promoter with corresponding terminator sequences. The cells were treated with the oxidant 4,4’‑dithiopyridine and lysates were subjected to immunoprecipitation with anti-FLAG beads, followed by non-reducing SDS-PAGE and immunoblotting with FLAG antibodies.

(**F**) Control for the experiment in **Fig. 2E**. The samples were treated with 50 mM DTT prior SDS-PAGE and immunoblotting.

(**G**) Der1-HA-I72C was co-expressed with either Hrd1-FLAG-I91C or Hrd1-FLAG-I91C-KRK in *hrd1∆der1∆* cells from CEN plasmids under the endogenous promoters. The cells were treated with the oxidant 4,4’‑dithiopyridine and lysates were subjected to immunoprecipitation with anti-FLAG beads, followed by non-reducing SDS-PAGE and immunoblotting with HA or FLAG antibodies.

**Figure S3. Systematic disulfide crosslinking of substrate and Der1.**

(**A**) Control for the experiment in **Fig. 3B.** The samples were treated with 50 mM DTT prior SDS-PAGE and immunoblotting.

(**B**) Der1-HA-F153C was expressed in *hrd1∆der1∆* or *der1∆* cells together with sCPY*-DHFR-Myc-C23, both from CEN plasmids under the endogenous promoters. The cells were treated with the oxidant 4,4’‑dithiopyridine and lysates were subjected to immunoprecipitation with anti-HA beads, followed by non-reducing (left side) or reducing (50 mM DTT, right side) SDS-PAGE and immunoblotting with HA and Myc antibodies.

(**C**) sCPY*-DHFR-Myc with single cysteines at different positions was co-expressed with Der1-HA constructs containing a single cysteine introduced at the indicated positions. Both substrate and Der1-HA were expressed from CEN plasmids in *der1∆* cells. The cells were treated with the oxidant 4,4’‑dithiopyridine and lysates were subjected to immunoprecipitation with anti-HA antibody beads, followed by non-reducing SDS-PAGE and immunoblotting with Myc antibodies.

**Figure S4.** **Probing Hrd3-substrate interactions by disulfide crosslinking.**

(**A**) Control for the experiment in **Fig. 5A**. The samples were treated with 50 mM DTT prior SDS-PAGE and immunoblotting.

(**B**) Control for the experiment in **Fig. 5C**. The samples were treated with 50 mM DTT prior SDS-PAGE and immunoblotting.
