# Supplementary material for: Disulfide-crosslink analysis of the ubiquitin ligase Hrd1 complex during endoplasmic reticulum-associated protein degradation
Source: J Biol Chem. 2022 Aug 13;298(9):102373. doi: 10.1016/j.jbc.2022.102373 (PMC9478403; doi:10.1016/j.jbc.2022.102373)
Supplement: Supplemental Figure S1 [file mmc2.pdf]

Figure S1

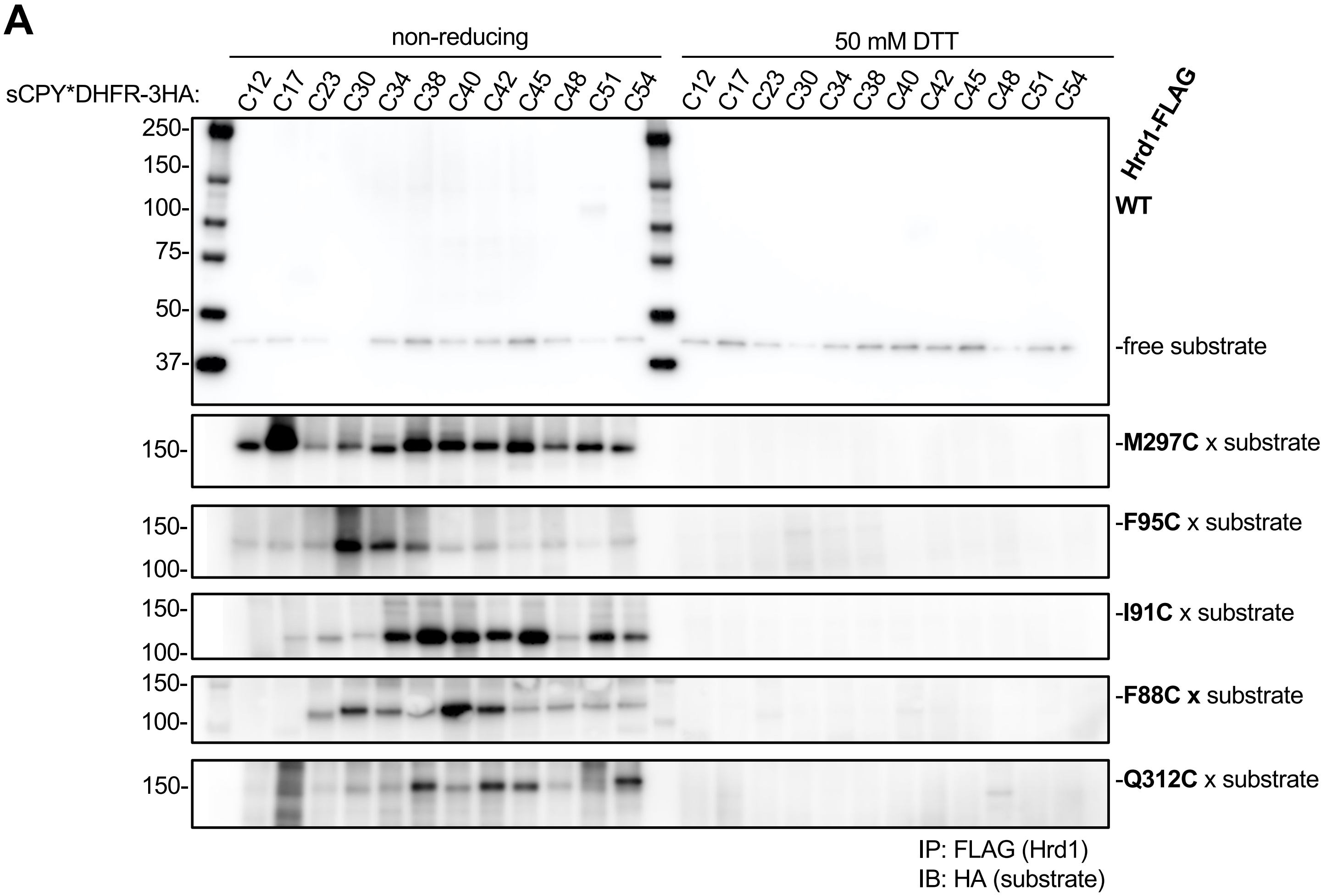
A
non-reducing
50 mM DTT
sCPY*DHFR-3HA:
C12 C17 C23 C30 C34 C38 C40 C42 C45 C48 C51 C54 C12 C17 C23 C30 C34 C38 C40 C42 C45 C48 C51 C54
Hrd1-FLAG
250-
150-
100-
75-
50-
37-
WT
-free substrate
150-
-M297C x substrate
150-
100-
-F95C x substrate
150-
100-
-I91C x substrate
150-
100-
-F88C x substrate
150-
-Q312C x substrate
IP: FLAG (Hrd1)
IB: HA (substrate)

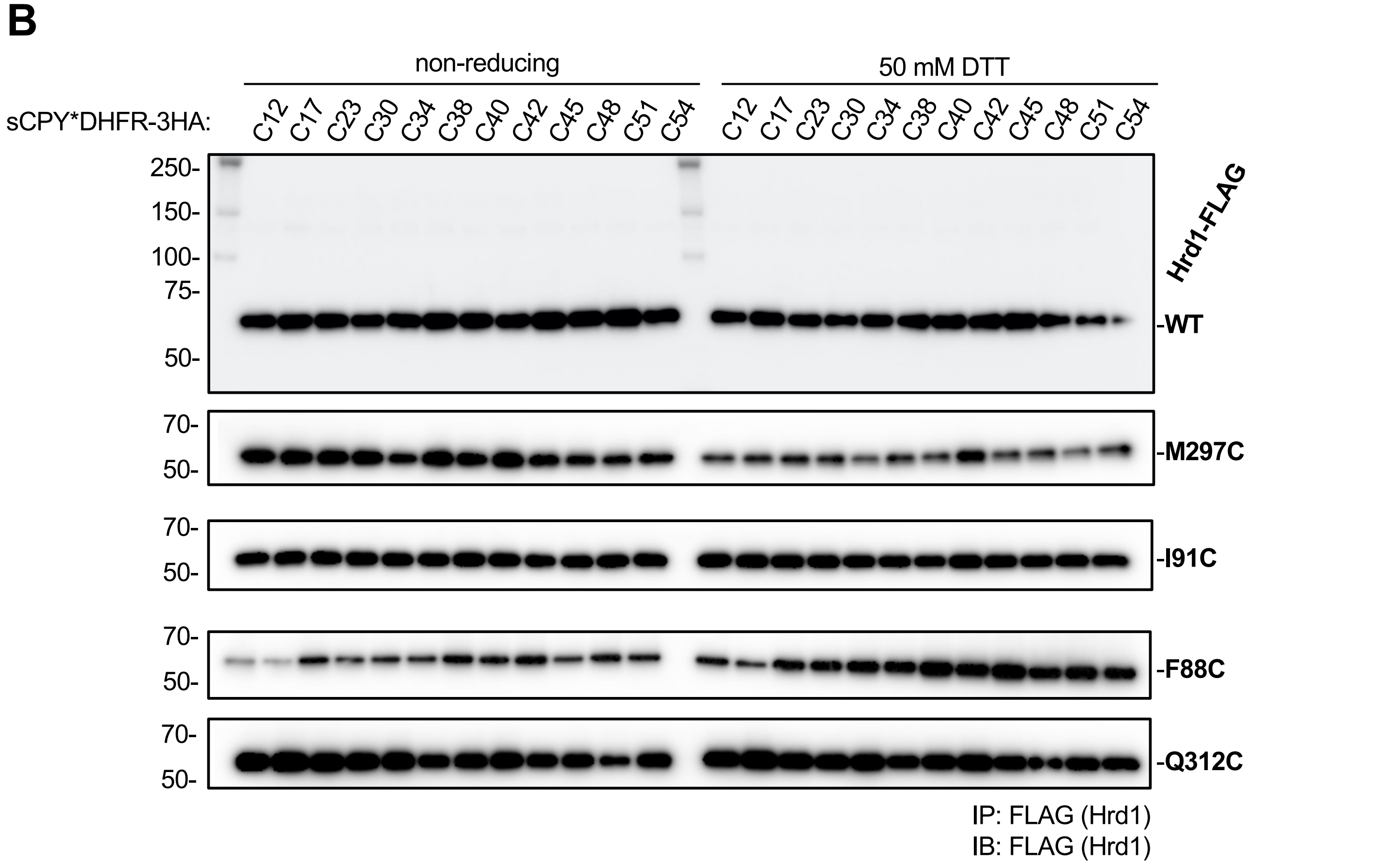
B
non-reducing
50 mM DTT
sCPY*DHFR-3HA:
C12 C17 C23 C30 C34 C38 C40 C42 C45 C48 C51 C54 C12 C17 C23 C30 C34 C38 C40 C42 C45 C48 C51 C54
Hrd1-FLAG
250-
150-
100-
75-
50-
-WT
70-
50-
-M297C
70-
50-
-I91C
70-
50-
-F88C
70-
50-
-Q312C
IP: FLAG (Hrd1)
IB: FLAG (Hrd1)

Figure S1

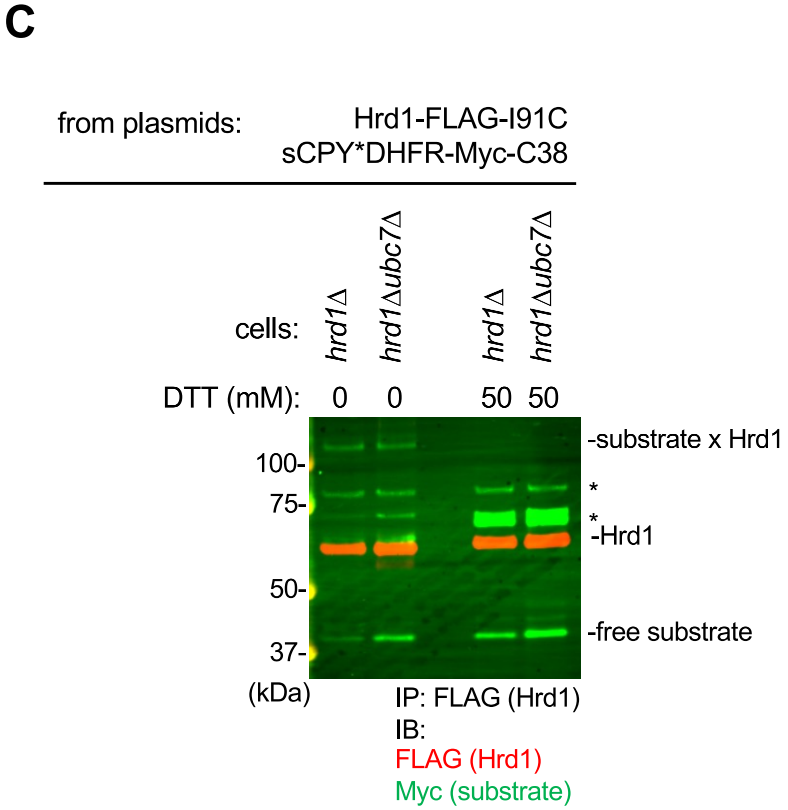
C
from plasmids:
Hrd1-FLAG-I91C
sCPY*DHFR-Myc-C38
cells: hrd1Δ hrd1Δubc7Δ hrd1Δ hrd1Δubc7Δ
DTT (mM): 0 0 50 50
100-
75-
50-
37-
(kDa)
-substrate x Hrd1
*
*
-Hrd1
-free substrate
IP: FLAG (Hrd1)
IB:
FLAG (Hrd1)
Myc (substrate)

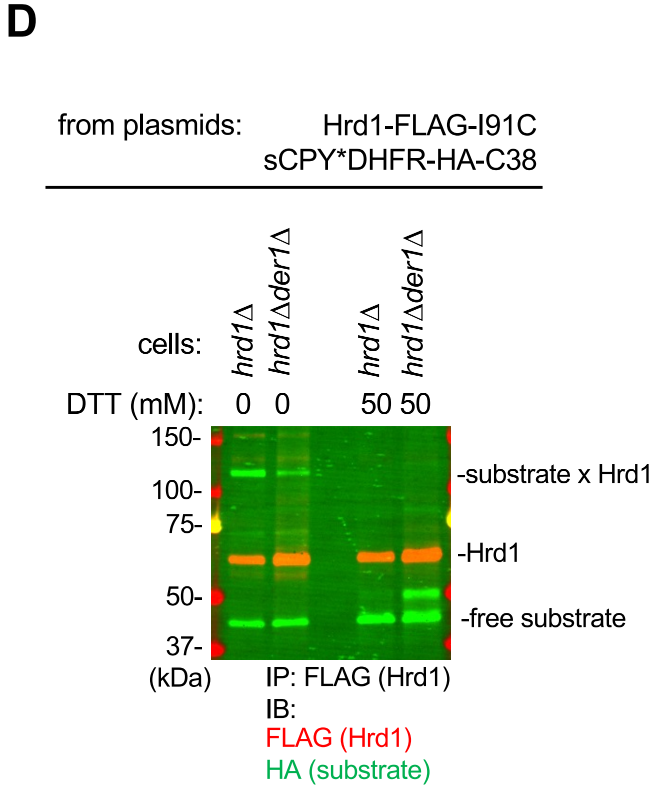
D
from plasmids:
Hrd1-FLAG-I91C
sCPY*DHFR-HA-C38
cells: hrd1Δ hrd1Δder1Δ hrd1Δ hrd1Δder1Δ
DTT (mM): 0 0 50 50
150-
100-
75-
50-
37-
(kDa)
-substrate x Hrd1
-Hrd1
-free substrate
IP: FLAG (Hrd1)
IB:
FLAG (Hrd1)
HA (substrate)

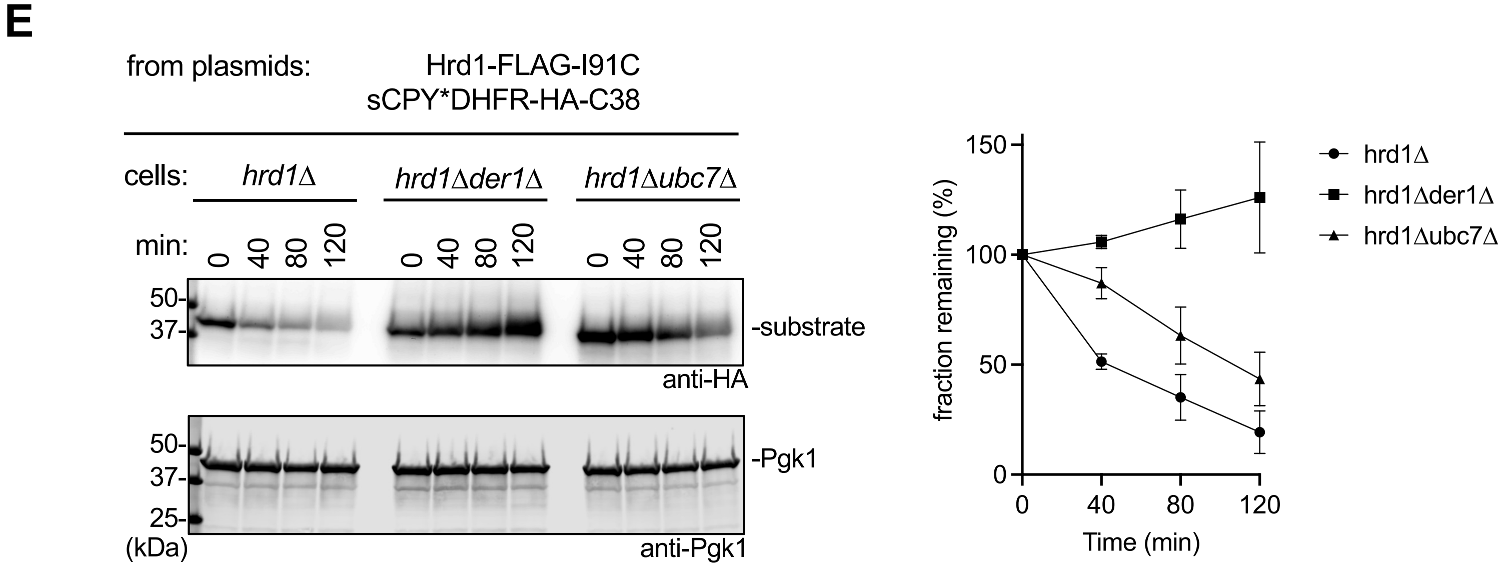
E
from plasmids:
Hrd1-FLAG-I91C
sCPY*DHFR-HA-C38
cells: hrd1Δ hrd1Δder1Δ hrd1Δubc7Δ
min: 0 40 80 120 0 40 80 120 0 40 80 120
50-
37-
-substrate
anti-HA
50-
37-
25-
(kDa)
-Pgk1
anti-Pgk1
150
100
50
0
fraction remaining (%)
0 40 80 120
Time (min)
hrd1Δ
hrd1Δder1Δ
hrd1Δubc7Δ
