# Supplementary figures and images for: Disulfide-crosslink analysis of the ubiquitin ligase Hrd1 complex during endoplasmic reticulum-associated protein degradation
Source: J Biol Chem. 2022 Aug 13;298(9):102373. doi: 10.1016/j.jbc.2022.102373 (PMC9478403; doi:10.1016/j.jbc.2022.102373)

Figure S3

A

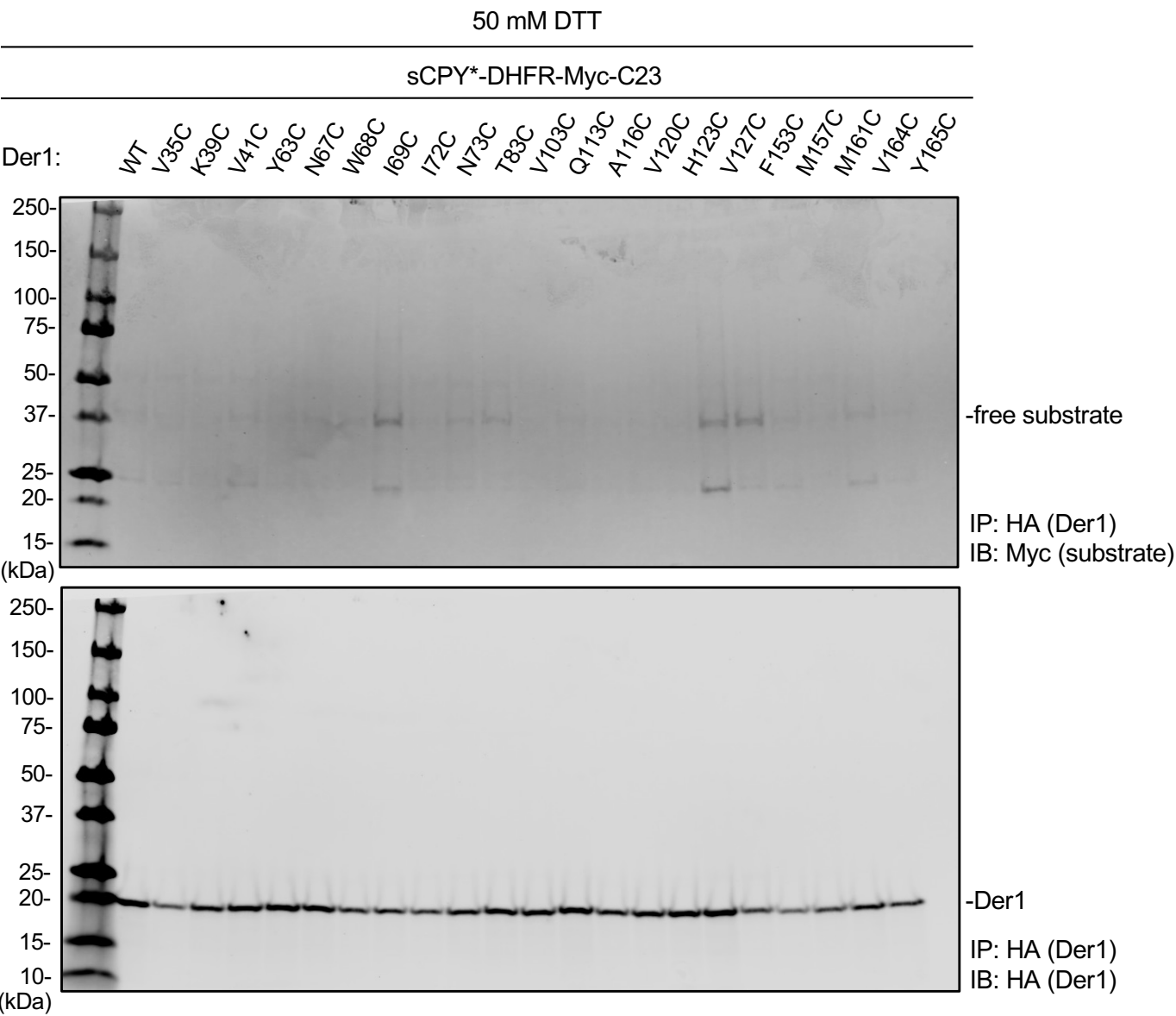

B

from plasmids: Der1-HA-F153C  
sCPY\*-DHFR-Myc-C23

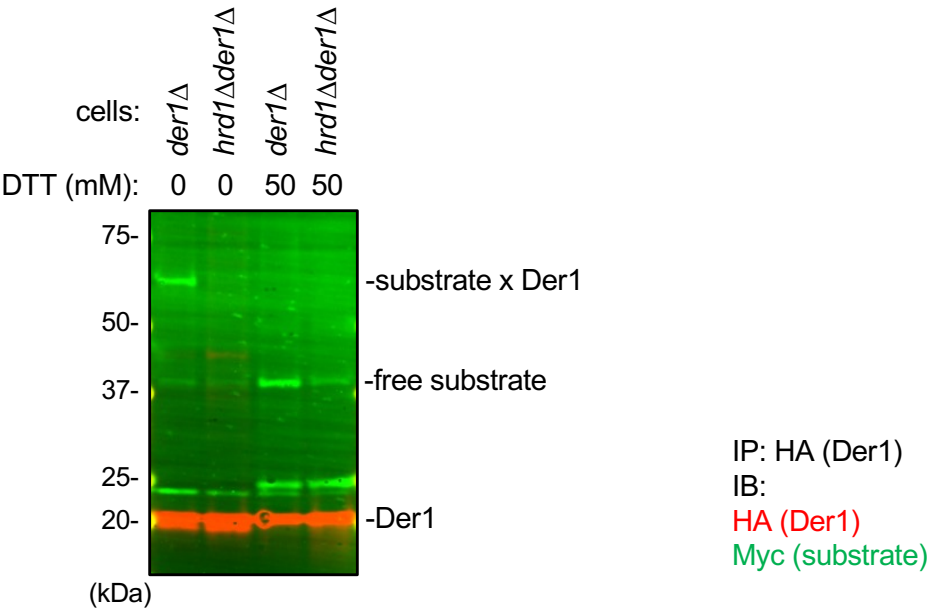

Figure S3  
C

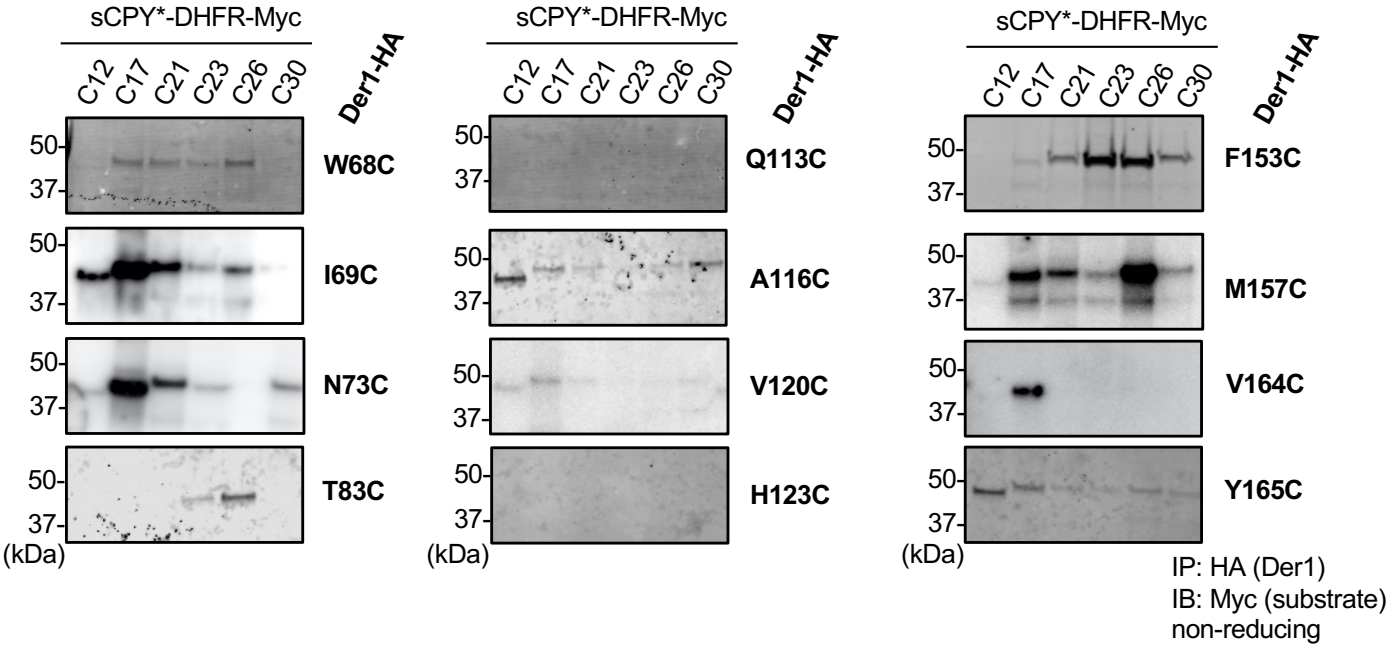

Supplement: Supplemental Figure S3 [file mmc3.pdf]

Figure S2

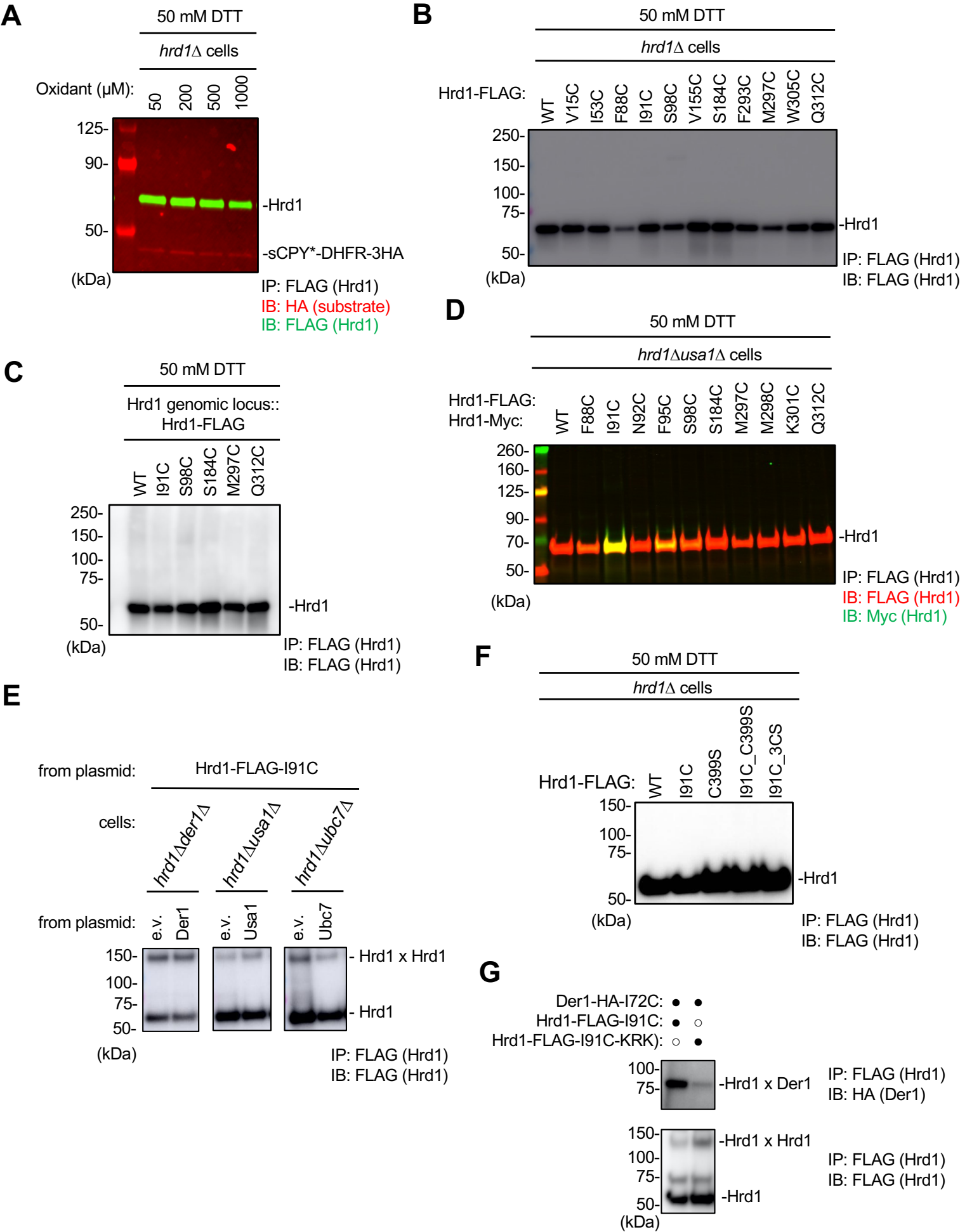

Supplement: Supplemental Figure S2 [file mmc4.pdf]

Figure S4

A

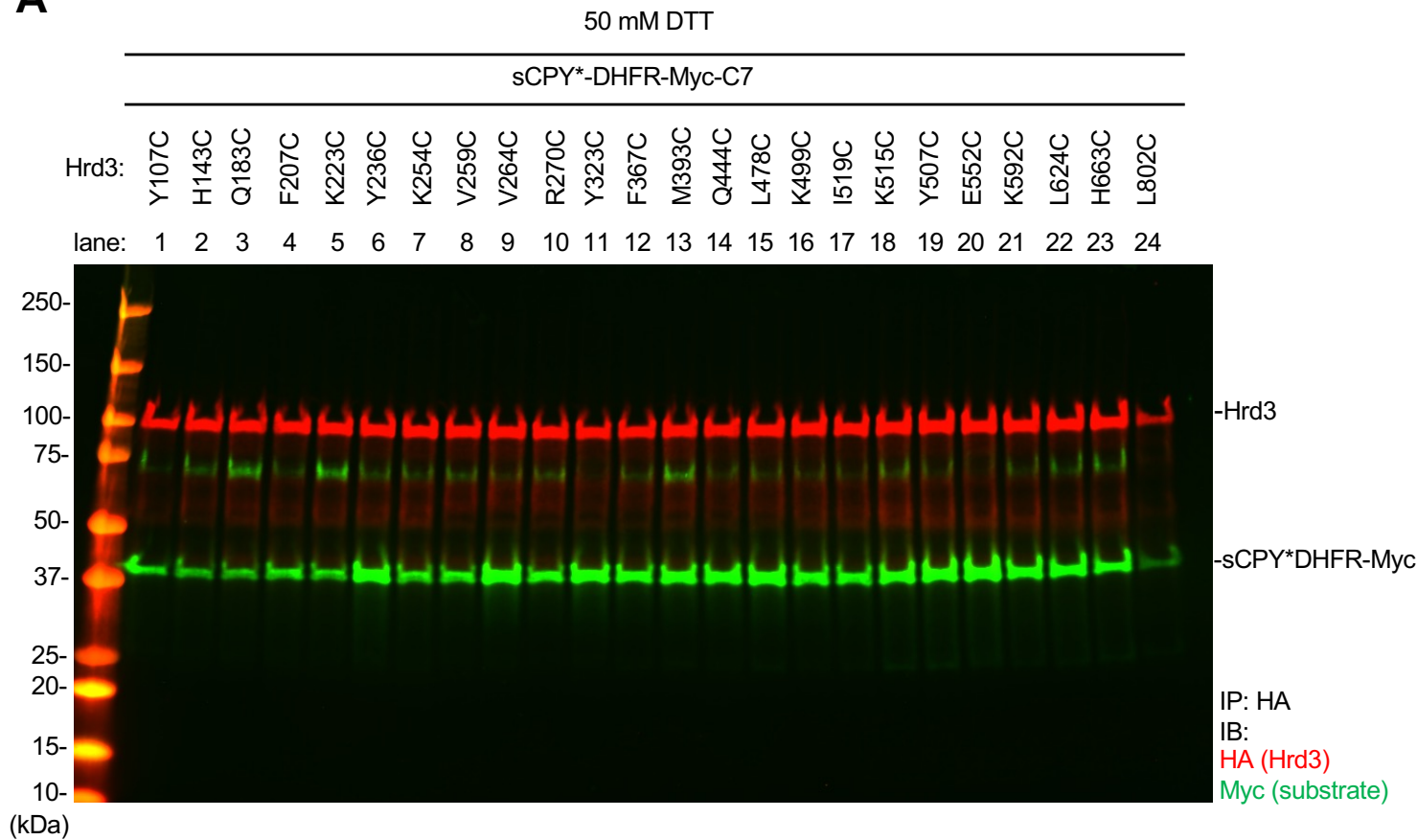

B

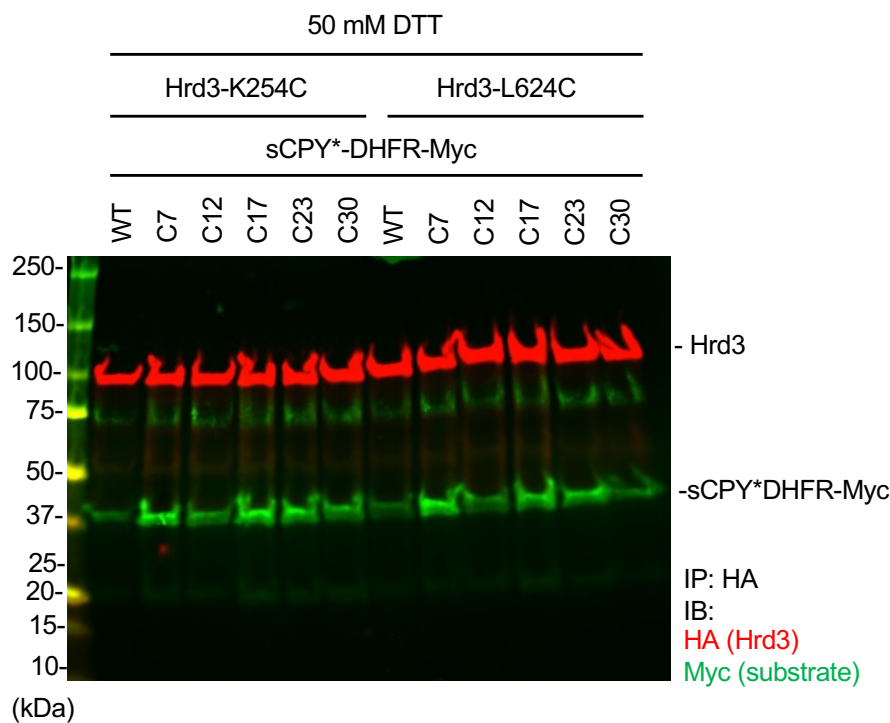

Supplement: Supplemental Figure S4 [file mmc5.pdf]
